# Amputation: Circular and Flap Operations

**Published:** 1829-03

**Authors:** 


19. *Amputation; Circular and Flap Operations.*—In No. 5, vol. II. of the Bost. Med. and Surg. Jour. we find the report of a case of *Fungus hœmatodes*, in which the thigh was amputated, by the distinguished Professor Warren. At the end of the article, the reporter takes the opportunity of stating the comparative estimate, in which the circular and the flap operations, are held, in the Massachusetts General Hospital. Cherishing the highest respect for the skill and science of Dr. W. we transcribe the following paragraph, for the information of our practical readers:

The circular and the flap operation of the thigh have been tried alternatively at the Hospital for some time back. On a review of these operations, the preference is given to the circular, for the following reasons:—1st. The wound made in this mode is not so vast. 2d. The hemorrhage is less. 3d. The incisions are more regular and precise, than in the flap operation. 4th. The constitutional affection, and, of course the danger is less in the circular, than the flap operation. The last can be done quicker, and makes a good stump. These advantages do not overbalance the objections. Whether the operation is a minute and a half, or four minutes in duration, is not so important as that the patient's safety should be ensured. When the operation is done, as a above described, all the incisions are smooth and exact; not the least irregularity of muscle appears on the face of the stump; and the covering of muscle, celular membrane, and skin, is as perfect as possible.

